# 35 Pharmacologic management of children with juvenile idiopathic arthritis in Batna, Algeria

**DOI:** 10.1093/rheumatology/keac496.031

**Published:** 2022-10-07

**Authors:** Hadef, S Slimani, M. C Khamari, W Mekaoussi, A Belot, P Quartier

**Affiliations:** 1 Department of Pediatrics, University Hospital Center of Batna, Faculty of Medicine, Batna 2 University; 2 Atlas Clinic of Rheumatology; 3 Private Clinic of Rheumatology, Batna, Algeria; 4 Pediatric Nephrology, Rheumatology, Dermatology Unit, Hôpital Femme Mère Enfant, Hospices Civils de Lyon, National Referee Centre for Rheumatic and AutoImmune and Systemic diseases in children (RAISE), Lyon, France; 5 Pediatric Immunology-Hematology and Rheumatology Unit Necker Hospital, Assistance Publique Hôpitaux de Paris, National Referee Centre for Rheumatic and AutoImmune and Systemic Diseases in Children (RAISE), Paris, France

## Abstract

**Background:**

Juvenile idiopathic arthritis (JIA) is the most common chronic rheumatic disease in childhood and is usually treated with non-steroidal anti-inflammatory drugs or disease-modifying anti-rheumatic drugs. The outcome in patients with JIA has markedly improved with the advent of biologic drugs.

**Objectives:**

The aim of this study was to describe treatments prescribed for children with JIA in Batna, Algeria.

**Methods:**

A multicentre retrospective and descriptive study was conducted in Batna health centers (public and private sectors), over a seven-year period from January 2013 to December 2019, based on (JIA patient’s data collection). As public sector source, we referred to the department of pediatrics of the university hospital center (CHU Benflis Touhami Batna), and as private sector source, we referred to private adult rheumatologists based in Batna.

**Results:**

The study included a total of 69 cases of JIA that were being followed in Batna health centers over the study period. Treatment modalities used for these patients included non-steroidal anti-inflammatory drugs (NSAIDs) in 54 patients (79.4%), steroids (prednisolone) in 37 patients (54.4%), conventional disease-modifying anti-rheumatic drug (c-DMARDs) in 51 patients (72.5%), biologic agents in 11 (15.9%) and intra articular injections in 17 patients (24.6%). The most frequently used c-DMARDs was methotrexate 42 (63.7%). The mean maximal dose reached was 7.5 mg (range 2.5–15). It was associated to other c-DMARDs in 2 cases. Sulfasalazine was used in 8 cases, Leflunomide in 1 case, and Hydroxychloroquine in 1 case. Biologics were used in 11 cases (15.9%).

RF-positive polyarthritis 4(50%), RF-negative polyarthritis 3 (33.3), systemic arthritis 2 (33.3) were the groups that most commonly needed a biological therapy. Biologics included Rituximab (*n* = 2), Tocilizumab (*n* = 1), Anakinra (*n* = 1), Etanercept (*n* = 1) and Infliximab (*n* = 1). The (mean duration of the disease evolution at the initiation of the biological agent) was 4.8 ± 5.4 years (range 0.5–8 years). At the time of enrolment, 31 patients (44.9%) were in remission: 20 patients (29%) were under treatment and 11 patients (15.9%) were not, while 12 patients (17.4%) had active disease.

**Conclusion:**

A high proportion of children presenting with JIA received cDMARDs. Biologics were needed in a few cases.

**Disclosure of Interest:**

None declared

